# Changes in GLP-1 and GIP During Endurance Training in Competitive Triathletes: A Pilot Observational Study

**DOI:** 10.3390/jcm15135069

**Published:** 2026-06-29

**Authors:** Michał Wiciński, Oskar Kuźmiński, Kamila Konopacka, Wiktor Kowalka, Witold Słomko, Marcin Gierach

**Affiliations:** 1 Department of Pharmacology and Therapeutics, Faculty of Medicine, Collegium Medicum in Bydgoszcz, Nicolaus Copernicus University, M. Curie 9, 85-090 Bydgoszcz, Poland; michal.wicinski@cm.umk.pl (M.W.); kamila.konopacka@cm.umk.pl (K.K.); wiktor.kowalka@cm.umk.pl (W.K.); 2 Department of Physiotherapy Collegium Medicum in Bydgoszcz, Nicolaus Copernicus University in Torun, 87-100 Toruń, Poland; witek.slomko@cm.umk.pl; 3 Department of Endocrinology and Diabetology Collegium Medicum in Bydgoszcz, Nicolaus Copernicus University in Torun, 87-100 Toruń, Poland; marcin77g@cm.umk.pl

**Keywords:** triathlon, hormones, incretins, testosterone, NF-κB

## Abstract

**Background:** Professional triathlon training is widely recognized as one of the most physiologically demanding forms of training, combining the multicomponent development of physical, biomechanical, and neuromuscular capacities across the three disciplines of swimming, cycling, and running. In this research, the authors measured the impact of two different training periods in elite male athletes on the serum concentrations of GLP-1, GIP, testosterone, and NF-κB and assessed their potential correlations. **Methods:** We compared a group of 37 triathletes, aged between 25 and 50, during preparatory and competitive periods, with a group of 20 healthy untrained males. The aim of the study was to assess the concentrations of testosterone, GLP-1, GIP, and NF-κB in both groups and in different exercise periods. We determined the markers using the ELISA method. **Results:** The results demonstrated an increase in GLP-1 concentration between two training groups (preparatory 44.73 ± 5.34 (pmol/L) vs. competitive 45.92 ± 6.13 (pmol/L); *p* < 0.001). Moreover, higher values of GIP serum concentration were observed in the training groups compared to the control group (33.21 ± 3.54 (ng/mL) vs. 38.83 ± 4.5 (ng/mL); *p* = 0.038). There were no statistically significant differences (*p* > 0.05) between the groups in terms of testosterone and NF-κB concentrations. There was also a statistically significant, strong positive correlation between NF-κB and GLP-1, GIP during each training period. In spite of the absence of definite changes in mean values of testosterone concentration between the groups, a minor increase in the median value in the group during the competitive period and a significantly lower median value in the control group were detected, suggesting a trend toward statistical significance. **Conclusions:** These findings may have clinical significance in athletes, as incretins and NF-κB could serve as biomarkers of metabolic adaptation to exercise and support individual training optimization. Further research is needed to investigate these relationships.

## 1. Introduction

Despite the growing interest in exercise-induced metabolic adaptations, limited data are available on the molecular adaptations of incretins, NF-kB, and testosterone associated with intensive endurance training in highly trained athletes. Similar studies have been conducted, but on considerably different groups of people, which is important because older or obese people have a divergent metabolism and hormonal response. Most previous studies examined the acute effect of a single bout of exercise, included recreationally active or obese individuals, or included people with an average age of over 60 years or already suffering from diabetes [1,2,3,4].

To the best of our knowledge, no previous study has simultaneously evaluated GLP-1, GIP, testosterone, and NF-κB concentrations across distinct training periods in elite male triathletes. Therefore, the aim of this study was to assess serum concentrations of GLP-1, GIP, testosterone, and NF-κB in male triathletes during two different training periods and to investigate the potential relationships between these parameters. We hypothesized that metabolic stress specific to the training phase influences incretin secretion and its association with hormonal and inflammatory markers. Moreover, the group of patients is not accidental and unique in the literature; competitive triathletes represent a unique model of extreme adaptive endurance, characterized by prolonged metabolic demand, significant carbohydrate metabolism, and repeated exposure to high physiological stress. This group of patients, as well as those training for ultratriathlons, is an ideal subject for studying the metabolic adaptations of highly athletic individuals. It is also important to note that the growing popularity of triathlon highlights the increasing clinical and scientific relevance of studies conducted in this athletic population.

## 2. Biological Mechanisms and Correlations Between Assessed Parameters

### 2.1. Structure, Function, and Levels of Testosterone

Testosterone is the primary androgen in the male body. Most of the testosterone, over 95%, is produced in the testes, specifically within the Leydig cells. During the process of steroidogenesis, cholesterol is the initial substrate from which pregnenolone is originally synthesized. Subsequently, there are two possible pathways for testosterone production: Δ4 and Δ5. In both pathways, the final step involves the enzymatic conversion by 17β-hydroxysteroid dehydrogenase, which converts androstenedione into testosterone [5].

Testosterone exerts several important effects in the human body that are relevant to this analysis. Firstly, it has an inhibitory impact on osteoclastic bone resorption and promotes osteogenesis by stimulating osteoblasts, thereby improving bone structure [6,7]. Additionally, testosterone stimulates muscle mass building and is associated with physical strength. In the central nervous system, it contributes to the enhancement of cognitive functions, and in the bone marrow, it stimulates erythropoiesis. It also influences sexual function, including erectile function and libido [8].

Testosterone levels vary across the population and show certain correlations. Studies, such as those conducted by Adi Z. et al., have demonstrated that individuals who train for a long time have higher testosterone concentrations compared to people with a sedentary lifestyle [9].

### 2.2. Structure, Function, and Levels of GLP-1, GIP, and NF-kB

Both glucagon-like peptide-1 (GLP-1) and glucose-dependent insulinotropic polypeptide (GIP) belong to the incretin hormone group and are largely responsible for postprandial insulin secretion. This phenomenon is called the incretin effect, which accounts for 50–70% of insulin secretion following oral administration of glucose. GLP-1 is a peptide produced mainly in the small intestine and colon in the L cells and in the brain. After food intake, the secretion of GLP-1 is mediated by sodium-glucose co-transporter 1 (SGLT1) [10], whereas GIP is produced mainly in the small intestine in the enteroendocrine K cells [2,11]. GLP-1 and GIP levels are not only dependent on food intake. A meta-analysis study conducted by Nejati R. et al. showed that GLP-1 levels also increase after exercise [12]. GLP-1 and GIP have mostly similar effects on tissues, except for their effect on glucagon secretion. GIP stimulates glucagon secretion, while GLP-1 inhibits it. The broad spectrum of incretins’ effects is worth paying attention to. GIP increases fat storage and stimulates pancreatic beta cell growth, inhibiting their apoptosis, and reduces bone resorption, whereas GLP-1 reduces gastric emptying, reduces appetite, stimulates pancreatic beta cell growth, and causes weight loss [13,14].

GLP-1 receptors are also found on the testes, but the influence of incretins on the reproductive system, including testosterone levels, is poorly researched. A study conducted by Jeibmann et al. showed that both glucose and GLP-1 decreased the frequency of testosterone pulses, implying that this suppression occurs by a mechanism that does not involve luteinizing hormone (LH) [15,16].

The nuclear factor kappa B (NF-κB) is a transcriptional regulator that plays a crucial role in various cellular processes, including the regulation of immune and inflammatory responses. It promotes the expression of various genes encoding, for example, cytokines, chemokines, and immune receptors. In the cell, NF-kB exists in the form of a complex with its inhibitor of kappa B (IkB) and therefore circulates in an inactive form in the cytoplasm waiting for an extracellular signal [17]. One of the cytokines stimulated for production by NF-kB is interleukin-6 (IL-6). Activation of this particular cytokine plays a key role in some inflammatory changes (like in rheumatoid arthritis, inflammatory bowel diseases, or thyroid autoimmune diseases) and neoplastic proliferation [18].

Physical exercise is one of the activators of NF-kB. This is associated, among other things, with muscle microdamage. NF-kB promotes increased protein turnover and regenerative muscle remodeling. It also adapts muscles to the oxidative stress of exercise. Physical exercise also increases IL-6 levels, which may also be related to increased NF-kB activity. IL-6 activity is thought to modulate glucose and lipid metabolism [19].

### 2.3. Correlation Between Testosterone, NF-κB, and Training

Testosterone is the most important anabolic hormone that promotes muscle growth and strength adaptations. However, physical activity has no significant effect on basal testosterone levels in trained men. The majority of the human studies assessing the interplay between exercise and basal testosterone have focused on adolescent men [20] or report only on acute responses. Data suggest that resistance training did not change basal testosterone levels in older men. In contrast, aerobic and interval training led to minor but significant increases in basal testosterone levels [21]. Certain studies even showed a reduction in resting testosterone levels during training, without any decline in physical performance [22]. In a study where the impact of endurance training was measured, the authors also observed a decrease in testosterone serum concentration as a possible effect of overtraining, higher catabolic activity, and stress factors triggered by under-recovery [23].

The primary role of transcription factor NF-κB signaling is to regulate innate immunity by responding to invading pathogens and harmful agents to promote gene expression, cytokine production, and cell survival under stressors [24]. Training induces a significant inflammatory response. NF-κB is a regulator of this response to exercise and has been shown to be activated after acute resistance exercise, mediating expression of pro-inflammatory cytokines such as TNF-*α*, IL-1β, and IL-6 [25]. After a single bout of intensive resistance training, upregulation of NF-κB occurs in human skeletal muscles. The research showed specific κB binding sites on the promoter region of genes that code MCP-1, IL-6, and IL-8. The binding to that region takes place 2 h post-exercise and returns to basal levels by 4 h [26].

Numerous studies have shown a significant negative correlation between testosterone and inflammatory markers (leptin, IL-6, TNF-*α*, MCP-1, and resistin) [27]. Higher testosterone levels after intensive physical training can lower IL-6 production [28]. Already, higher levels of testosterone in active athletes can strengthen the effects. There is experimental evidence that activation of the androgen receptor by dihydrotestosterone can inhibit TNF-*α* or LPS-induced NF-κB p65 nuclear translocation and reduce expression of COX2 and pro-inflammatory cytokines in human benign prostatic hyperplasia cells [29].

## 3. Training Characteristics in Professional Triathlon

Professional triathlon training is structured around multicomponent, periodized programming aimed at the concurrent development of physiological, biomechanical, and neuromuscular capacities across the three disciplines of swimming, cycling, and running [30]. A typical training week consists of 18 to 30 h of structured physical activity, organized into 9 to 12 distinct sessions. Training distribution is modulated according to the athlete’s event specialization, individual physiological profile, and phase of the competitive season. Modality-specific volume and intensity are adjusted to optimize performance while minimizing the risk of overtraining and injury.

Training content includes a combination of extensive low-intensity endurance work (Zone 1–2), threshold training (Zone 3), and high-intensity interval training targeting VO_2_max (Zone 4–5), alongside discipline-specific technique drills and brick sessions that simulate race transitions. Resistance training (2–3 times per week) is implemented primarily during the general preparation phase to support neuromuscular robustness and injury prevention [31].

The annual training cycle is typically divided into three primary phases: preparatory (general and specific), competitive, and transitional [32]. During the general preparatory phase, the emphasis is placed on building aerobic capacity, general strength, and technical efficiency. The specific preparatory phase introduces higher training intensities and race-specific simulation. The competitive phase is marked by a reduction in training volume and a shift toward race-pace sessions, tapering, and active recovery. The transitional phase serves as a period of physical and psychological regeneration before the onset of a new macrocycle.

## 4. Materials and Methods

### 4.1. Bioethics

The study was conducted in alignment with the standards established by the Declaration of Helsinki. Informed consent forms were obtained in writing from all participants in advance of the beginning of the study. The ethics committee of Collegium Medicum in Bydgoszcz, Nicolaus Copernicus University, in Toruń approved the research protocol and design (approval number: KB 407/2023, approval date: 26 September 2023).

### 4.2. Study Cohort

**Figure 1:**
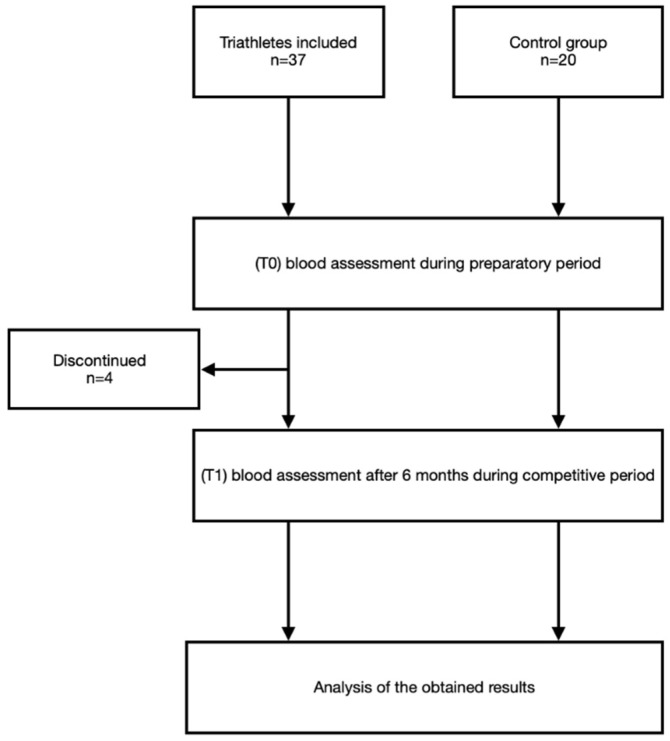
Patient flowchart for observational study.

The authors collected 37 samples for examination of the level of specific human hormones: testosterone, GLP-1, GIP, and NF-κB. The study was conducted during two periods of personalized triathlete training. The blood samples were collected during the preparatory (time point 0, group 1) and competitive (time point 1, group 2) periods. The interval between time points 1 and 2 was approximately 6 months (Figure 1). Since the main aim of the study was to analyze biomarker adaptation in a highly trained group of healthy triathletes, all of the participants had no prior medical history of chronic disease and were non-smokers, maintaining their status throughout the duration of the study. The athletes were aged between 25 and 50, with active participation in triathlon competition for a minimum of 4 years (practicing 6–7 times weekly, 6 to 13 training sessions). A triathlete’s weekly regimen contains 6 to 13 training sessions each week, which, in addition to swimming, cycling, and running, also include strength training. The athletes focus on improving one component at a time—running, swimming, or cycling skills—at different points throughout the season. The control group consists of 20 healthy males who do not participate in triathlons or any other sports, with similar anthropometric characteristics to the study group (BMI between 18.5 and 24.9 kg/m^2^). The primary exclusion criteria included the use of any medications and any substances that may be considered an illegal enhancement of one’s physical abilities; however, none of the participants had a history of usage of such substances.

### 4.3. Measurements

The anthropometric characteristics were provided by the Department of Pharmacology and Therapeutics, Faculty of Medicine, Collegium Medicum in Bydgoszcz. Venous blood samples of the participants were collected before and after a specified period of training. The participants were asked to remain in a fasted state (with the last meal scheduled to be in the evening the day before the blood collection). The samples were drawn between 9 AM and 10 AM in a laboratory environment.

The serum was prepared following a standard laboratory procedure, frozen at −20 °C, and transported on dry ice to a central facility, where it was stored at −70 °C until analysis. Assays were performed using stringent quality control. Biomarkers were determined using the ELISA method on a BioTek EPOCH instrument (BioTek, Winooski, VT, USA).

### 4.4. Statistical Analysis

Data are presented as mean values with standard error of the mean (±SEM) and additional minimum and maximum values. In order to check the compliance of the results distribution with the normal distribution for the results obtained during the preparatory period (time point 0) and the competitive period (time point 1), the Shapiro–Wilk test was used. For dependent samples (i.e., study group before vs. study group after) without a normal distribution, the Wilcoxon signed-rank test was used. For independent samples without a normal distribution, the Mann–Whitney U test was used to compare the participants in two training periods with the control group. Finally, correlations were established using the Spearman rank correlation test.

## 5. Results

**Figure 2:**
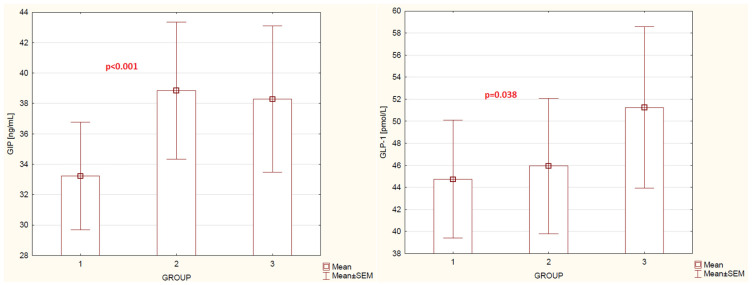
Mean ± SEM serum concentrations of GLP-1 and GIP in each group during the (1) preparatory and (2) competitive periods and (3) a control group (*p* < 0.05—group 1 vs. group 2).

Mean ± SEM values for testosterone, GLP-1, GIP, and NF-κB concentrations were calculated for each group, with 95% confidence intervals (CIs). Apparent differences in GLP-1 concentration with statistical significance were noticed between the two training groups (preparatory 44.73 ± 5.34 (pmol/L) vs. competitive 45.92 ± 6.13 (pmol/L); *p* < 0.001). Simultaneously, the control group showed increased GLP-1 levels. Results also showed higher values of serum GIP concentration in the competitive group vs. the preparatory group (33.21 ± 3.54 (ng/mL) vs. 38.83 ± 4.5 (ng/mL); *p* = 0.038) (Table 1). Furthermore, the research demonstrated that GIP concentration between the non-training group and the training groups is invariably higher (Figure 2).

**Figure 3:**
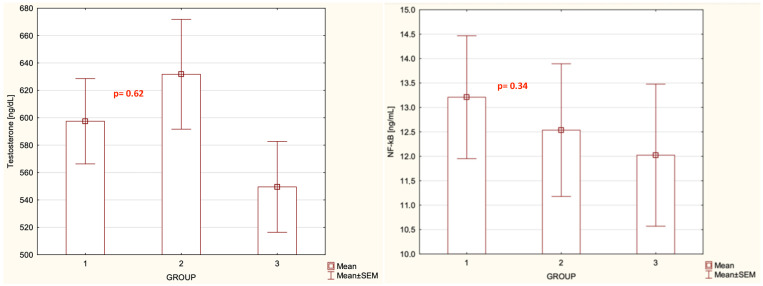
Mean ± SEM serum concentrations of testosterone and NF-κB in each group during the (1) preparatory and (2) competitive periods and (3) a control group (*p* > 0.05 for all comparisons).

In the study, there were no statistically significant differences (*p* > 0.05) between the groups in terms of testosterone and NF-κB concentrations. Despite the absence of definite alteration in mean values of testosterone concentration between the groups, we observed a slight increase in the median value in group 2 and a significantly lower median value in group 3 (control) (Figure 3).

**Figure 4:**
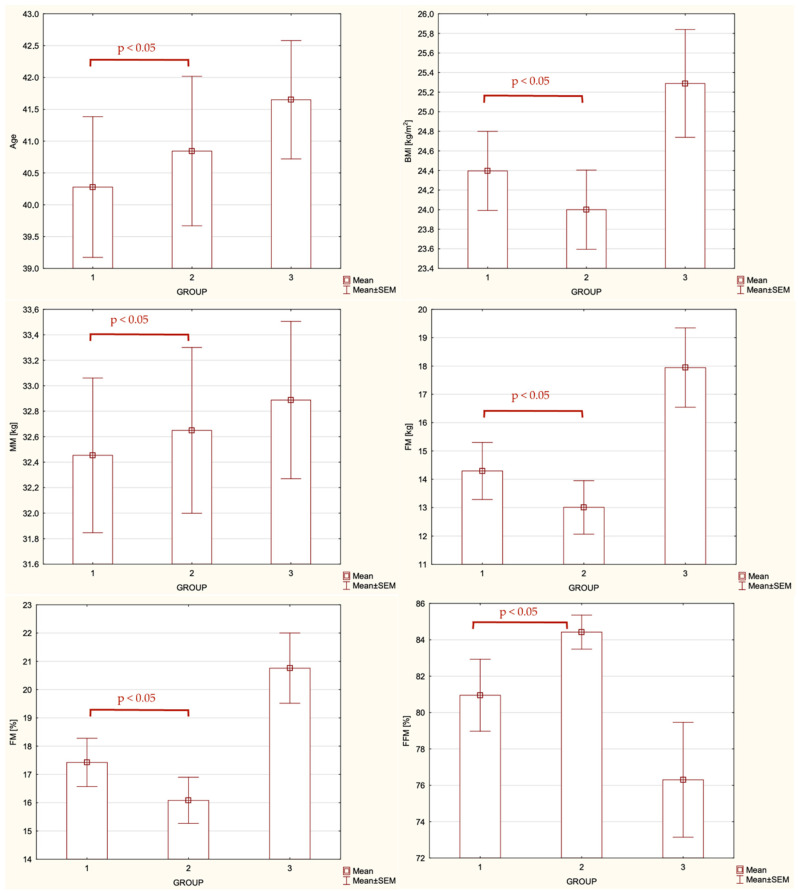
Mean ± SEM measurements of age, muscle mass (MM), body mass index (BMI), fat mass (FM), and fat-free mass (FFM) in each group during the (1) preparatory and (2) competitive periods and (3) a control group (*p* < 0.05 for comparisons between groups 1 and 2).

The authors also provide values for the following parameters: age, muscle mass (MM), body mass index (BMI), fat mass (FM), and fat-free mass (FFM). Measurements were performed in individual groups during three observation periods: (1) the preparatory period, (2) the competition period, and (3) in the control group. Statistical significance was observed in the results between groups 1 and 2 (Table 2, Figure 4).

**Figure 5:**
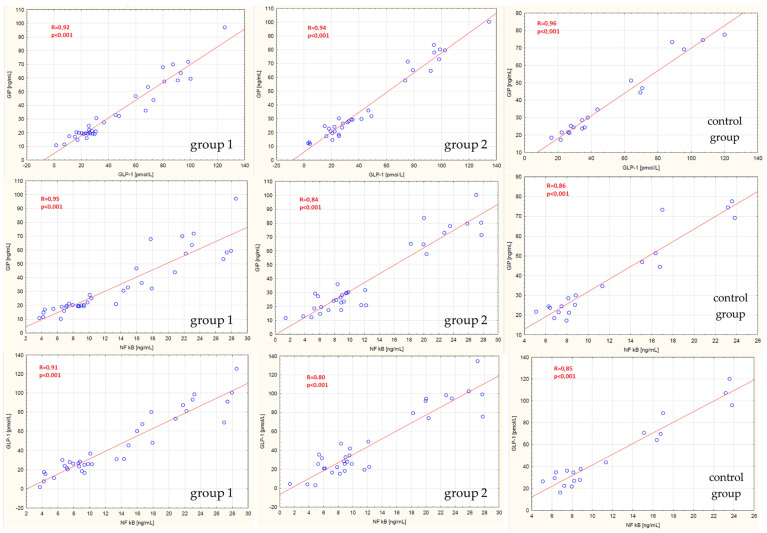
Positive correlation between NF-κB and GLP-1 and GIP during each training period (*p* < 0.05).

Additionally, the authors assessed whether there was a correlation between GLP-1, GIP, and NF-κB. The analyses demonstrated a statistically significant, strong positive correlation between NF-κB and GLP-1 and GIP during each training period (Figure 5).

## 6. Discussion

The first aspect considered in this study is lower GLP-1 in trained individuals, which might be caused by the fact that endurance training improves GLP-1 sensitivity, thus lowering the baseline levels of GLP-1. Conceivably, enhanced responsiveness of beta cells to GLP-1 could downregulate GLP-1 release from L cells [33]. These results were consistent with those reported in the study by Janus et al. that showed a 20% drop in fasting GLP-1 levels for every hour of moderate-intensity physical activity performed [34]. Conversely, lower GIP will result in lower GLP-1. The correlation between GLP-1 and GIP in our study may occur via the mechanism described by Timper et al., where GIP stimulates pancreatic alpha cells to produce IL-6, which in turn stimulates the production of GLP-1 [35]. In an investigation of the regulatory mechanisms of GLP-1 secretion, GIP was demonstrated to be the most efficacious stimulator for GLP-1 release [36]. A very strong positive correlation of GLP-1 and GIP as incretin hormones further supports the close physiological relation and dependency. The high concordance of both hormone concentrations before observation may suggest well-preserved and coordinated gut–pancreatic function. Moreover, these results might show that sustained endurance training supports the preservation of the appropriate incretin response. In contrast, some studies show that glucocorticoids decrease the secretion of GLP-1 in L cells; consequently, an increase in cortisol after physical activity might lead to lower levels of GLP-1 in trained individuals [3].

**Figure 6:**
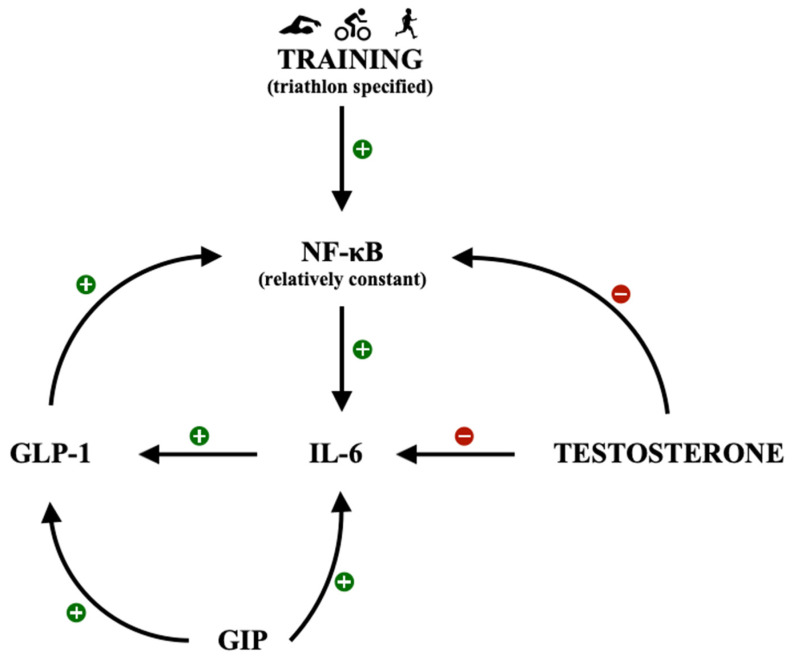
Possible mechanisms explaining the correlation between the discussed parameters.

Our study found that GIP levels were lower in the preparatory period. This finding correlates with the study by Vasto et al., which showed a rise in GIP levels after 20 weeks of the SuperJump program [4]. A positive correlation was also found between NF-kB and incretins, and this relationship is based on the mediating effect of IL-6 [37]. NF-kB is a key mediator of IL-6 activation. It acts through both direct and indirect mechanisms, e.g., through interleukin-1 (IL-1) and tumor necrosis factor alpha (TNF-alpha) [38,39]. Furthermore, IL-6 stimulates GLP-1 release after exercise. Without IL-6 stimulation, postprandial and postexercise GLP-1 levels were lower in the study conducted by Ellingsgaard et al. [40]. Previously, the same conclusions were drawn by the same researcher in a study on mice [41]. Moreover, it can be postulated that a positive feedback loop may exist that further amplifies this stimulation, as GLP-1 has been shown to activate NF-κB signaling in pancreatic β-cells [42] (Figure 6).

In our study, the NF-kB level is relatively constant in all groups, which may be due to several reasons. Firstly, the cause may be that the activation of NF-κB was short-lived. NF-kB transiently increases after exercise and then normalizes. Normalization occurs after 4 h [18]. However, in the study by Durham W. et al., NF-kB was examined by muscle biopsy; its level decreased immediately after exercise, but after an hour of rest, it returned to normal. This return to pre-exercise NF-kB levels within a short time appears in both studies [43]. Similar data were also obtained by Gallego-Selles A. et al. In this study, the level of NF-kB was checked before and after heavy exercise in the biopsied muscle in a group of eleven active men. The study demonstrated that maintaining blood flow through the muscle results in decreasing the level of NF-kB just one minute after exercise [44]. Of particular importance is the fact that, in elite athletes, NF-κB activation may represent the physiological adaptive response to high-intensity training, rather than a sign of a pathological inflammatory state. Improved insulin sensitivity, improved glucose metabolism, and prospective attenuation of an excessive inflammatory response may all be compensatory effects of the elevated GLP-1 concentration.

In the case of testosterone, a study conducted by Tremblay et al. showed that the increase in serum concentration depends on the type of exercise. After endurance exercise, which also includes triathlon, the increase is smaller than in resistance exercise [45]. When interpreting the results, it is important to consider that a study by Jensen et al. has demonstrated a transient increase in testosterone levels after exercise. This elevation is short-lived, with concentrations returning to baseline after about two hours of recovery [46]. Analyzing both studies, it is worth noting that the intensity probably influences changes in testosterone levels more than the type of exercise. Another study by Kraemer et al. showed a drop to baseline testosterone levels after an initial increase just 15 min after the end of exercise [47].

The observed changes in incretin levels may reflect broader metabolic adaptations related to exercise, body composition, and inflammatory regulation. Some systematic reviews have shown that interventions influencing metabolic pathways, including vitamin D and probiotic supplementation, can influence body weight, insulin sensitivity (including influencing GLP-1 levels), inflammation, and metabolic homeostasis [48,49].

Although our study did not focus on changes in the gut microbiota, diet, and supplementation of participants, it is important to consider the connection between incretins and the composition of the gut microbiota. According to new research, the gut microbiome may play a key role in controlling incretin secretion and metabolic adaptation. Moreover, recent research suggests a reciprocal relationship between gut microbiota composition and GLP-1 signaling [50]. Firstly, gut microbiota metabolites stimulate GLP-1 secretion and affect GLP-1 function and rhythm, whereas the mechanism of action of GLP-1 on gut microbiota involves the inflammatory response [51]. The release of GLP-1 from intestinal L-cells may be influenced by gut-derived inflammatory pathways, bile acid signaling, and short-chain fatty acids (SCFAs). Additionally, endurance training itself may alter the intestinal flora, which could be a factor in the metabolic changes seen in highly skilled athletes [52]. It is worth noting that some studies showed that nausea, vomiting, diarrhea, pancreatitis, bowel obstruction, and notably small intestinal bacterial overgrowth (SIBO) may be linked to increased GLP-1 receptor stimulation [53].

As expected, statistical analysis of BMI, body weight, and fat mass comparing the study groups showed statistical significance. Physical activity promotes fat burning and muscle building. The lower GLP-1 in the study group also confirms and aligns with these results. A study conducted on a group of 1326 individuals showed that physical activity was associated with lower fasting GLP-1 values and a higher postprandial release in men. This promotes better glucose-insulin metabolism [34]. Consequently, it reduces fat mass—in the case of the study by Åkerström T. et al., visceral fat tissue—and probably also reduces the regain of body weight [33,54].

## 7. Limitations

Our study has several limitations that should be considered. The sample size was relatively modest; however, it was sufficient to detect the primary effects. Additionally, it is comparable to those used in cohorts in similar physiological studies.

The homogenous characteristics of the study participants enhance reliability but may limit the generalizability of the findings to other athletic populations involved in different types of activity. Moreover, the assessment of testosterone, GLP-1, GIP, and NF-κB was restricted to selected time points, although these windows correspond to relevant post-exercise periods identified in other studies.

The body composition was assessed using BMI, which may have limited interpretability in highly trained athletes due to increased lean body mass. Future studies should incorporate more precise body composition assessment methods, such as DXA or bioimpedance analysis.

The lack of objective training load quantification (e.g., Training Stress Score or intensity distribution), which limited our ability to directly associate endocrine changes with training stimuli. Additionally, potential confounding factors such as dietary intake, hydration status, supplementation, sleep recovery, and circadian hormonal variation were not systematically controlled and should be incorporated in future longitudinal studies involving endurance athletes.

Finally, the observational nature of the study does not allow for causal inference, yet the findings are consistent with established mechanistic models.

## 8. Conclusions

All our findings have potential clinical relevance, especially in the population professionally involved in sport. The fact that endurance training may increase GLP-1 sensitivity and lower baseline levels of GLP-1 may be the reason why our studies depict lower fasting GLP-1 concentrations in trained individuals. Overall, this could support future optimization of training and metabolic strategies for athletes. In the future, incretins may be an indicator of how the body adapts to training loads, and monitoring incretin levels might help to assess carbohydrate balance. Together, NF-kB and incretins may provide an answer to whether a person is having the correct training adaptation and allow for planning an optimal training regimen for each individual’s metabolic needs. In order to compare and contrast the findings and provide a better in-depth look into the mechanisms, further and more specific research should be performed.

## Figures and Tables

**Table 1:** Mean values of GLP-1, GIP, testosterone, and NF-κB serum concentrations in the analyzed groups.

		Group	
control group [3]	competitive period [2]	preparatory period [1]	
51.24 ± 7.32	45.93 ± 6.14	44.73 ± 5.34 *	GLP-1 [pmol/L]
38.29 ± 4.8	38.84 ± 4.5	33.21 ± 3.54 *	GIP [ng/mL]
549.24 ± 33.16	631.74 ± 40.1	597.47 ± 31.15	Testosterone [ng/dL]
12.02 ± 1.45	12.53 ± 1.36	13.21 ± 1.26	NF-κB [ng/mL]

All confidence intervals are reported at the 95% level. * *p* < 0.05—group during the preparatory period vs. group during the competitive period.

**Table 2:** Mean ± SEM measurements of age, body mass index (BMI), muscle mass (MM), fat mass (FM), and fat-free mass (FFM) in each group during the (1) preparatory and (2) competitive periods and (3) a control group (*p* < 0.05 for comparisons between groups 1 and 2).

		Group	
control group [3]	competitive period [2]	preparatory period [1]	
41.65 ± 0.9	40.84 ± 1.2	40.28 ± 1.1 *	Age
25.29 ± 0.6	24.0 ± 0.4	24.40 ± 0.4 *	BMI
32.9 ± 0.6	32.65 ± 0.65	32.45 ± 0.6 *	MM [kg]
17.9 ± 1.4	12.53 ± 1.36	14.29 ± 1.0 *	FM [kg]
20.8 ± 1.2	13.0 ± 0.9	17.43 ± 0.9 *	FM [%]
76.3 ± 3.2	84.42 ± 0.9	80.95 ± 2.0 *	FFM [%]

All confidence intervals are reported at the 95% level. * *p* < 0.05—group during the preparatory period vs. group during the competitive period.

## Data Availability

The data presented in this research are available upon request from the corresponding author.

## Abbreviations

The following abbreviations are used in this manuscript:

GLP-1	Glucagon-like peptide-1
GIP	Glucose-dependent insulinotropic polypeptide
SGLT1	Sodium-glucose co-transporter 1
NF-κB	The nuclear factor kappa B
IkB	Inhibitor of kappa B
IL-6	Interleukin-6
TNF-α	Tumor necrosis factor alpha
IL-1β	Interleukin-1 beta
MCP-1	Monocyte chemoattractant protein-1
LPS	Lipopolysaccharide
COX2	Cyclooxygenase-2
VO_2_max	Maximal oxygen uptake
